# Dissolved Organic Carbon Regulates Bacterial Ingestion by *Tetraselmis* sp.

**DOI:** 10.1007/s00248-026-02752-z

**Published:** 2026-04-07

**Authors:** Tzu-Hao Chiang, Sagaya John Paul J, Yun-Chi Lin, Kuo-Ping Chiang

**Affiliations:** 1 Institute of Marine Environment and Ecology, National Taiwan Ocean University, Keelung, Taiwan; 2 General Education Center, National Taiwan Ocean University, Keelung, Taiwan; 3 Taiwan Ocean Genome Center, National Taiwan Ocean University, Keelung, Taiwan; 4 Center of Excellence for the Oceans, National Taiwan Ocean University, Keelung, Taiwan

**Keywords:** Biolog PM1, Glucose, Mixotrophy, Osmotrophy

## Abstract

*Tetraselmis* sp. is a widespread marine green alga capable of both dissolved organic carbon (DOC) and bacterial ingestion, making it a potentially important contributor to carbon fluxes in microbial food webs. Mixotrophy can enhance survival under nutrient or light limitation, yet how DOC availability influences phagotrophic activity in marine green algae remains poorly understood. Here, we experimentally examined the interaction between osmotrophy and phagotrophy in *Tetraselmis* sp. under low-light and dark conditions across different dissolved organic carbon levels. Bacterial ingestion rates were quantified using live fluorescently labeled bacteria (FLB), enabling direct assessment of feeding responses. Carbon utilization assays showed that *Tetraselmis* sp. can assimilate a broad range of organic substrates, with glucose preferentially utilized under low-light conditions. Increasing glucose concentrations (0.1, 1, and 10 g L⁻¹) significantly suppressed bacterial ingestion, with ingestion rates declining from 2.34 to 0.17 bacteria cell⁻¹ h⁻¹ under low-light conditions and from 1.48 to 0.16 bacteria cell⁻¹ h⁻¹ in darkness. Together, these results demonstrate that DOC availability directly regulates phagocytosis, suggesting a trade-off between osmotrophic uptake and phagotrophic feeding. Our findings refine current frameworks of algal mixotrophy by highlighting the interaction among multiple trophic strategies and underscore the trophic plasticity of marine green algae in shaping carbon transfer within microbial food webs.

**Supplementary Information:**

The online version contains supplementary material available at 10.1007/s00248-026-02752-z.

## Introduction

*Tetraselmis* is a motile green alga (Chlorodendrophyceae) widely distributed in coastal and estuarine waters, where it functions as a primary producer and occasionally forms localized blooms [1, 2]. Members of this genus tolerate broad ranges of salinity, temperature, and light, reflecting substantial ecological plasticity [3, 4]. Although *Tetraselmis* has been shown to utilize glucose [5] and is also capable of bacterial ingestion [6], the effect of DOC availability on phagotrophy has not been experimentally tested in marine eukaryotic algae.

Mixotrophy, defined as the coexistence of phototrophy and phagotrophy within a single cell [7], can enhance survival under fluctuating light and nutrient conditions [8], particularly under nutrient or light limitation [9–11]. However, maintaining multiple trophic pathways incurs energetic costs, implying trade-offs in resource allocation [9]. While osmotrophy is sometimes included within broader definitions of mixotrophy [10, 11], interactions between osmotrophic and phagotrophic pathways remain poorly understood, especially in marine systems [12]. Here, we test the hypothesis that dissolved organic carbon regulates phagotrophy in *Tetraselmis* sp. through a metabolic trade-off between osmotrophic uptake and bacterial ingestion.

## Materials and Methods

### Organic Carbon Utilization by *Tetraselmis* sp.

Biolog PM1 carbon source plates were used to assess organic carbon utilization by *Tetraselmis* sp. A “bacteria-only” control was prepared by filtering cultures through a 0.8 μm polycarbonate membrane. Experiments were conducted over 3 days with an initial algal density of ~ 1 × 10⁶ cells mL⁻¹ under low-nutrient conditions (K/20 medium) at 25 °C. Prior to the experiments, *Tetraselmis* cultures were maintained under a 12:12 h light: dark cycle. During the experiments, cultures were assigned to either a low-light treatment (10 ± 5 µE m⁻² s⁻¹, 12:12 h light: dark cycle) or a dark treatment, with the plates wrapped in aluminum foil. Each treatment was performed in a single experimental replicate.

Each well of the 96-well Biolog plate (95 carbon sources plus one blank, shown in Table S1) received 100 µL of algal suspension and Redox Dye D (1% final concentration), and plates were sealed to reduce evaporation. Absorbance was measured at 590 nm (metabolic activity) and 750 nm (cell density) using a microplate reader (Synergy™ Mx, BioTek). Carbon sources were considered utilizable when absorbance at 590 nm exceeded that of the blank. Algal metabolic activity was estimated by subtracting values of bacteria-only control from those of the algae-containing treatments.

### Bacterial Ingestion under Four Glucose Concentrations

To examine the effect of dissolved organic carbon on bacterial ingestion, *Tetraselmis* sp. was incubated with four glucose concentrations (0, 0.1, 1, and 10 g L⁻¹) under low-light or dark conditions for 4 days, with three biological replicates per treatment. On Day 4, 1 mL aliquots were collected to estimate ingestion rates using live fluorescently labeled bacteria (FLB) [13, 14]. Prior to each experiment, ambient bacterial abundance in each sample was quantified using flow cytometry, with concentrations ranging from approximately 3–16 × 10⁶ cells mL⁻¹. The bacterial prey consisted of a strain isolated from the algal culture and identified as *Staphylococcus saprophyticus*. The bacterium was initially cultured on solid Marine Broth 2216 agar plates and subsequently transferred to liquid Marine Broth 2216, where it was grown overnight at 37 °C. Bacterial cells were then stained with CellTracker™ Green CMFDA (10 µM) at 37 °C for 3 h, followed by three washes with artificial seawater to generate fluorescently labeled bacteria (FLB). FLB were then added to the samples, and bacterial abundance was quantified again to verify the proportion of added FLB. The amount of FLB added corresponded to approximately 20–30% of the ambient bacterial abundance, and incubations were conducted for 1 h. The saturation time of bacterial ingestion by Tetraselmis sp. is described in the Supplementary Materials and Figure S1.Additionally, 200 µL subsamples were stained with LysoTracker (50 nM), and the proportion of stained algal cells was determined by counting at least 100 cells under a fluorescence microscope.

##  Results and Discussion

### Biolog Analysis of Organic Carbon Utilization by *Tetraselmis*

**Fig. 1:**
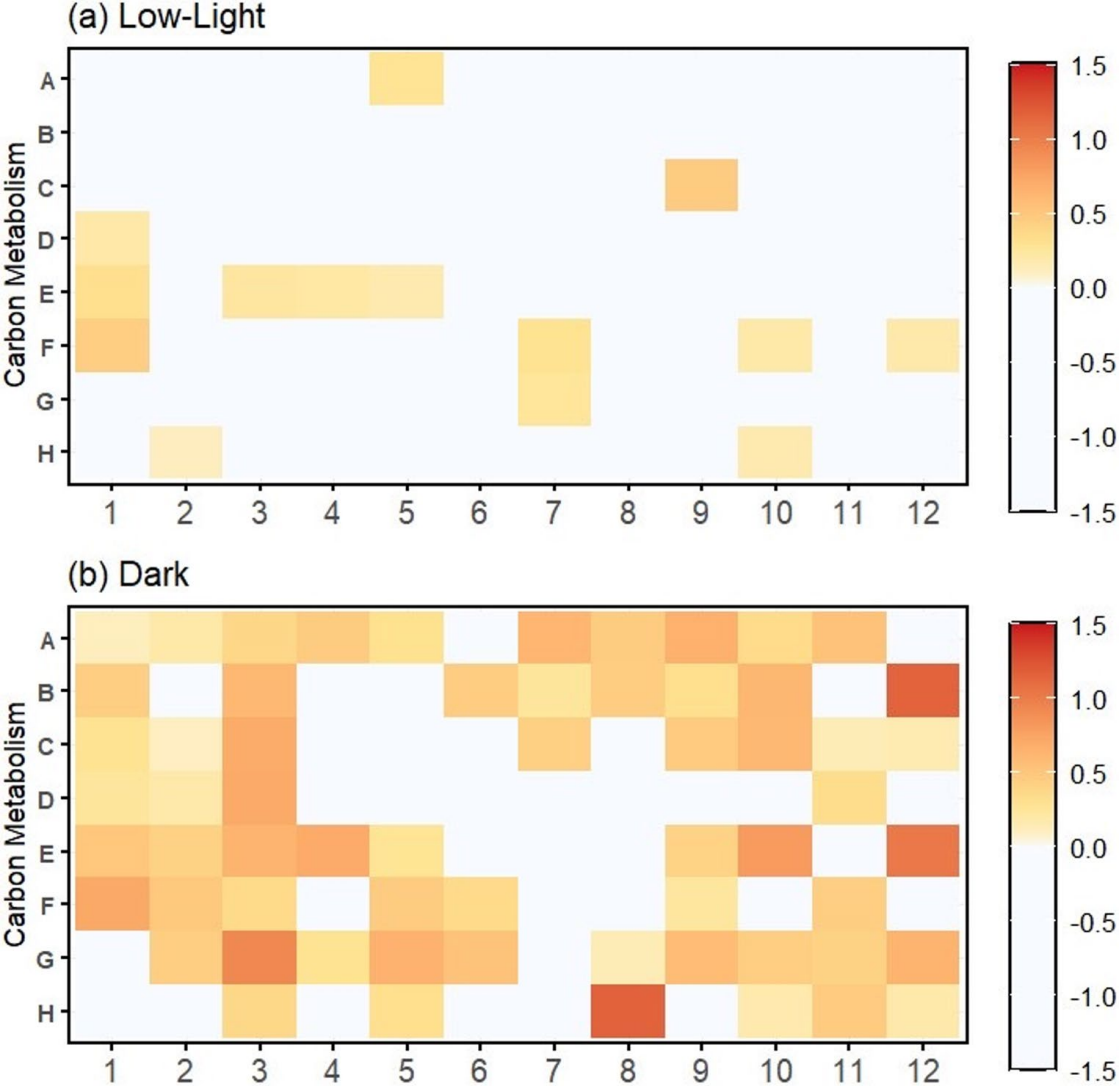
Carbon metabolic activity of *Tetraselmis* sp. under low-nutrient conditions measured using Biolog PM1 microplates. Metabolic responses to 95 different carbon substrates and one control were assessed under (**a**) low-light and (**b**) dark conditions. Values represent the “algae + bacteria” treatment after subtraction of the “bacteria-only” control, providing an estimate of algal carbon metabolic capacity. Under low-light conditions (**a**), the top five utilized substrates were α-D-glucose (0.475, C9), glycyl-L-aspartic acid (0.460, F1), L-glutamine (0.323, E1), propionic acid (0.294, F7), and succinic acid (0.290, A5). Under dark conditions (**b**), the top five substrates were pyruvic acid (1.172, H8), L-glutamic acid (1.166, B12), adenosine (1.044, E12), L-serine (0.940, G3), and maltotriose (0.824, E10). Individual carbon metabolic activity and corrected maximum daily growth rate (NRmax) for the “algae + bacteria” and “bacteria-only” treatments are shown in Figures S2 and S3

Biolog PM1 microplates were used to assess organic carbon utilization under low-light and dark conditions. To estimate algal-specific carbon utilization, signals from the “bacteria-only” treatment were subtracted from those of the “algae + bacteria” treatment. Individual carbon metabolic activity and corrected maximum daily growth rate (NRmax) for the “algae + bacteria” and “bacteria-only” treatments are shown in Figures S2 and S3.Although Biolog PM1 plates were originally developed for bacterial metabolic profiling, they have increasingly been applied to microalgae to assess heterotrophic carbon use [15, 16]. Under low-light conditions, α-D-glucose showed the highest utilization (Fig. 1a), suggesting preferential use of carbohydrate-associated substrates, potentially supported by residual photosynthetic energy. In contrast, under dark conditions, pyruvic acid exhibited the highest utilization (Fig. 1b), suggesting a shift toward respiratory metabolites. These patterns indicate light-dependent modulation of organic carbon metabolism, with low light supporting mixotrophic supplementation and darkness favoring respiratory maintenance.

### The Ingestion Rates under Different Glucose Concentrations

**Fig. 2:**
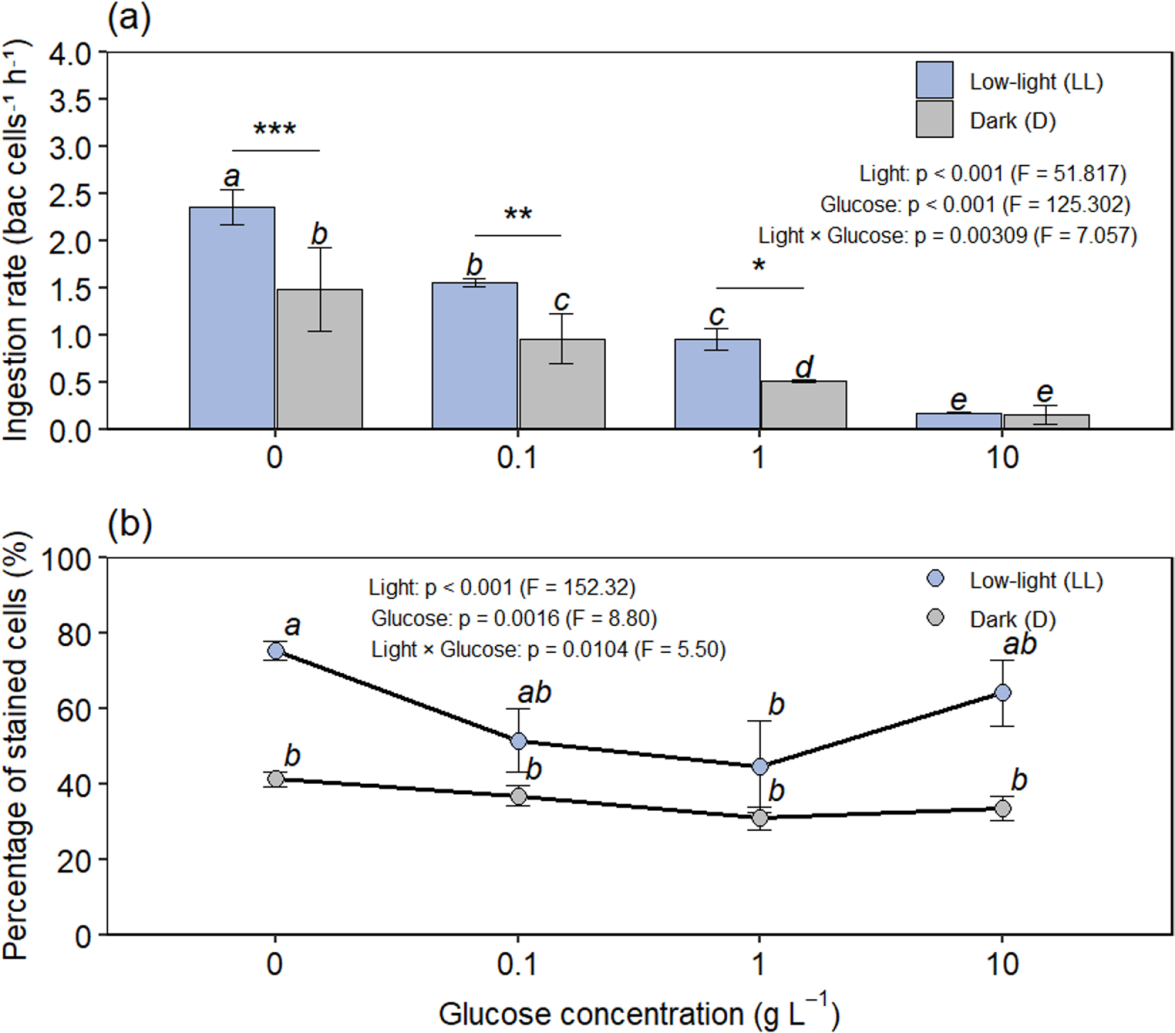
Ingestion rates and staining responses of *Tetraselmis* sp. under different light and glucose conditions. (**a**) Bar plots show the number of fluorescently labeled bacteria (FLB) ingested per algal cell per unit time across different glucose concentrations. (**b**) Line plots show the percentage of LysoTracker-stained cells per 100 algal cells. Glucose concentrations included four treatments: 0, 0.1, 1, and 10 g L⁻¹. Data represent means of triplicate experiments, and error bars indicate standard deviations. Ingestion rates were analyzed using two-way ANOVA followed by Tukey’s HSD post hoc test, with different letters indicating significant differences among treatments. Asterisks denote levels of statistical significance: *p* < 0.05, *p* < 0.01, and *p* < 0.001. Metabolic activity of glucose was shown in Figure S4

Four glucose concentrations (0, 0.1, 1, and 10 g L⁻¹) were tested under low light and darkness. Ingestion was highest without glucose and declined progressively with increasing glucose under both treatments (Fig. 2). Under low light, the ingestion rate was 2.34 bac cell⁻¹ h⁻¹ at 0 g L⁻¹ glucose and decreased to 1.56, 0.96, and 0.17 bac cell⁻¹ h⁻¹ as glucose concentration increased to 0.1, 1, and 10 g L⁻¹, respectively. Under dark conditions, ingestion rates declined from 1.48 bac cell⁻¹ h⁻¹ at 0 g L⁻¹ to 0.96, 0.51, and 0.16 bac cell⁻¹ h⁻¹ with increasing glucose concentration. Two-way ANOVA revealed significant effects of light (*p* < 0.001), glucose concentration (*p* < 0.001), and their interaction (*p* < 0.01). Post hoc tests (Tukey’s HSD) showed that ingestion at 0 g L⁻¹ was significantly higher than at all other concentrations under low light, with stepwise declines as glucose increased. Ingestion was also significantly higher under low light than darkness at 0–1 g L⁻¹, but not at 10 g L⁻¹, confirming a light × glucose interaction. Glucose uptake was likewise reduced under dark conditions (Figure S5), indicating that both particulate and dissolved carbon acquisition were constrained in the absence of light. The inverse relationship between ingestion and glucose concentration suggests regulatory substitution rather than simultaneous maximization of carbon acquisition pathways, consistent with energy-allocation trade-off models of mixotrophy.

It is important to note that the highest glucose concentration tested (10 g L⁻¹) exceeds typical marine DOC levels [17]. These concentrations were intentionally selected to span a broad range of carbon availability and to examine physiological responses under carbon-replete conditions rather than to directly mimic in situ DOC concentrations. The selected range was informed by previous laboratory studies [18]. In preliminary tests, higher glucose levels (25–100 g L⁻¹) did not induce additional metabolic responses (data not shown), suggesting that responses had approached saturation. Thus, the concentrations used here represent a gradient from carbon-limited to carbon-replete conditions.

LysoTracker staining was used to assess acidic food vacuole activity. Under low light, the percentage of positive cells peaked at 0 g L⁻¹ glucose (75%), declined at intermediate concentrations (51% and 44%), and increased again at 10 g L⁻¹ (64%). In contrast, under dark conditions, the proportion of positive cells remained relatively stable (31–41%). However, LysoTracker fluorescence did not correlate with ingestion rates (Figure S5, r² = 0.12, *p* = 0.42), consistent with known limitations of acidotropic dyes in quantitatively assessing phagotrophy [6, 19, 20]. In *Tetraselmis*, fluorescence likely reflects acidic compartments beyond food vacuoles.

Ingestion rates are commonly quantified using DTAF-stained bacteria [13]. However, prey selectivity varies among protistan taxa [21]. In preliminary experiments with *Tetraselmis* sp., no ingestion was detected when fluorescent beads (0.5–1 μm) or heat-killed, fluorescently stained bacteria were offered, whereas clear phagocytosis occurred when live bacteria were provided. Accordingly, all ingestion rate measurements in this study were conducted using live bacterial prey. This behavior is consistent with previous findings that some green algae preferentially ingest live bacteria [14].

Ingestion rates of *Tetraselmis* sp. (0.16–2.34 bacteria cell⁻¹ h⁻¹) peaked under low-light conditions without glucose and fall within the range reported for green algae (< 3 bacteria cell⁻¹ h⁻¹) [22, 23]. Because bacterivory regulation varies among taxa and environmental conditions [14, 22, 23], prey selectivity may influence carbon partitioning in microbial communities [24]. Preferential ingestion of specific bacteria could redirect carbon flow toward algal biomass and reduce competition for dissolved organic carbon. Overall, these findings highlight taxon-specific and condition-dependent control of bacterivory within chlorophytes.

## Conclusion

This study demonstrates that dissolved organic carbon availability regulates phagotrophy in *Tetraselmis* sp. Glucose suppressed bacterial ingestion, indicating that organic carbon directly influences trophic strategy in this species. While carbon fluxes were not quantified, our results show that mixotrophic behavior is sensitive to resource availability. Incorporating organic carbon dynamics into conceptual models may improve our understanding of trophic regulation in marine microbial systems.

## Supplementary Information

Below is the link to the electronic supplementary material.

Supplementary Material 1

## Data Availability

The data that support the findings of this study are available from the corresponding author upon reasonable request.
