# Supplementary material for: Dissolved Organic Carbon Regulates Bacterial Ingestion by Tetraselmis sp
Source: Microb Ecol. 2026 Apr 7;89:106. doi: 10.1007/s00248-026-02752-z (PMC13171760; doi:10.1007/s00248-026-02752-z)
Supplement: Supplementary file 1 [file 248_2026_2752_MOESM1_ESM.dotx]

**Supplemental Materials**

**Determine the saturation time of bacterial ingestion by *Tetraselmis* sp.**

To determine the ingestion saturation time of *Tetraselmis* sp., exponentially growing cells cultured in K medium were incubated with live, fluorescently labeled Staphylococcus saprophyticus. A 1 mL algal culture (1.03 × 10⁶ cells mL⁻¹) was mixed with fluorescent bacteria to a final concentration of 1.14 × 10⁷ FLB mL⁻¹ (approximately 10–30% of the original bacterial abundance). Experiments were conducted in triplicate, with observations every 15 min for 120 min. At each time point, ingested bacteria were counted in 100 algal cells, and ingestion rates were calculated at 15-min intervals. The ingestion saturation point was defined as the time of maximum ingestion rate.

**Saturation Time of Tetraselmis sp. Ingestion**

The results showed that the ingestion saturation time of Tetraselmis sp. was approximately 60 minutes. During the first 60 minutes, the ingestion rate steadily increased and peaked at 60 minutes, reaching approximately 0.19 FLB cell⁻¹ (Figure S4). The ingestion rate began to decline at 75 minutes and continued to decrease until 120 minutes, reaching its lowest value. Therefore, a 60-minute incubation period was used as the standard for calculating ingestion rates in subsequent experiments. However, unlike the commonly observed pattern in which ingestion rates plateau after reaching saturation, the decline observed in this study suggests that ingestion by Tetraselmis sp. is not continuously maintained after saturation.

**Supplemental Table**

Table S1. Layout of carbon substrates in the Biolog PM1 carbon utilization assay. The table lists the carbon substrate assigned to each well and its position within the 96-well plate.

| A1  Negative Control | A2  L-Arabinose | A3  N-Acetyl-D-  Glucosamine | A4  D-Saccharic Acid | A5  Succinic Acid | A6  D-Galactose | A7  L-Aspartic Acid | A8  L-Proline | A9  D-Alanine | A10  D-Trehalose | A11  D-Mannose | A12  Dulcitol |
| --- | --- | --- | --- | --- | --- | --- | --- | --- | --- | --- | --- |
| B1  D-Serine | B2  D-Sorbitol | B3  Glycerol | B4  L-Fucose | B5  D-GlucuronicAcid | B6  D-Gluconic Acid | B7  D,L-α-GlycerolPhosphate | B8  D-Xylose | B9  L-Lactic Acid | B10  Formic Acid | B11  D-Mannitol | B12  L-Glutamic Acid |
| C1  D-Glucose-6-Phosphate | C2  D-Galactonic  Acid-γ-Lactone | C3  D,L-Malic Acid | C4  D-Ribose | C5  Tween 20 | C6  L-Rhamnose | C7  D-Fructose | C8  Acetic Acid | C9  α-D-Glucose | C10  Maltose | C11  D-Melibiose | C12  Thymidine |
| D1  L-Asparagine | D2  D-Aspartic Acid | D3  D-Glucosaminic  Acid | D4  1,2-Propanediol | D5  Tween 40 | D6  α-Keto-GlutaricAcid | D7  α-Keto-ButyricAcid | D8  α-Methyl-DGalactoside | D9  α-D-Lactose | D10  Lactulose | D11  Sucrose | D12  Uridine |
| E1  L-Glutamine | E2  m-Tartaric Acid | E3  D-Glucose-1-Phosphate | E4  D-Fructose-6-Phosphate | E5  Tween 80 | E6  α-HydroxyGlutaric Acid-γ-  Lactone | E7  α-HydroxyButyric Acid | E8  ß-Methyl-D-Glucoside | E9  Adonitol | E10  Maltotriose | E11  2-Deoxy  Adenosine | E12  Adenosine |
| F1  Glycyl-L-Aspartic  Acid | F2  Citric Acid | F3  myo-Inositol | F4  D-Threonine | F5  Fumaric Acid | F6  Bromo Succinic Acid | F7  Propionic Acid | F8  Mucic Acid | F9  Glycolic Acid | F10  Glyoxylic Acid | F11  D-Cellobiose | F12  Inosine |
| G1  Glycyl-L-Glutamic  Acid | G2  Tricarballylic Acid | G3  L-Serine | G4  L-Threonine | G5  L-Alanine | G6  L-Alanyl-Glycine | G7  Acetoacetic Acid | G8  N-Acetyl-ß-D-Mannosamine | G9  Mono Methyl Succinate | G10  Methyl Pyruvate | G11  D-Malic Acid | G12  L-Malic Acid l |
| H1  Glycyl-L-Proline | H2  p-Hydroxy  Phenyl AceticAcid | H3  m-Hydroxy  Phenyl Acetic Acid | H4  Tyramine | H5  D-Psicose | H6  L-Lyxose | H7  Glucuronamide | H8  Pyruvic Acid | H9  L-Galactonic Acid-γ-Lactone | H10  D-Galacturonic Acid | H11  Phenyle-thylamine | H12  2-Aminoe-thanol |

**Supplemental Figures**

Figure S1. Ingestion of fluorescently labeled bacteria (FLB) by *Tetraselmis* sp. over time. The y-axis shows the mean number of FLB ingested per cell, and the x-axis indicates incubation time (min). Each symbol represents the mean of three replicate samples under the corresponding condition, with error bars indicating standard deviations. The solid line represents a logistic model fitted to the ingestion data, illustrating that FLB uptake approaches saturation after approximately 60 min.

Figure S2. Carbon metabolic activity of *Tetraselmis* sp. under low-nutrient conditions measured using Biolog PM1 microplates. Metabolic responses to 95 carbon substrates and one control were assessed under (a) low-light and (b) dark conditions. For each light treatment, heatmaps show results from the “algae + bacteria” and “bacteria-only” treatments. Metabolic activity was quantified based on absorbance measured at 590 nm.

Figure S3. Corrected maximum daily growth rate (NRmax) was calculated based on absorbance measured at 750 nm. NRmax responses to 95 carbon substrates and one control were assessed under (a) low-light and (b) dark conditions. For each light treatment, heatmaps show results from the “algae + bacteria” and “bacteria-only” treatments.

Figure S4. Changes in metabolic activity of *Tetraselmis* sp. under different glucose concentrations and light conditions. Metabolic activity is expressed as absorbance at 590 nm, reflecting the extent of cellular glucose utilization. Two light regimes were tested: (a) low light and (b) darkness, with four glucose concentrations (0, 0.1, 1, and 10 g L⁻¹) applied in each treatment. Data represent the mean of three replicates, and error bars indicate standard deviation.

Figure S5. Linear regression analysis between grazing rate and the percentage of LysoTracker-stained cells. Blue and gray points represent low-light (LL) and dark (D) conditions, respectively. The regression equation, R² value, and p value are shown in the figure. The shaded gray area indicates the 95% confidence interval.
